# Effect of Substrates Performance on the Microstructure and Properties of Phosphate Chemical Conversion Coatings on Metal Surfaces

**DOI:** 10.3390/molecules27196434

**Published:** 2022-09-29

**Authors:** Chunmiao Du, Kangqing Zuo, Zongliang Ma, Minru Zhao, Yibo Li, Shuai Tian, Yupeng Lu, Guiyong Xiao

**Affiliations:** 1 Key Laboratory for Liquid-Solid Structural Evolution and Processing of Materials, Ministry of Education, Shandong University, Jinan 250061, China; 2 School of Materials Science and Engineering, Shandong University, Jinan 250061, China

**Keywords:** phosphate chemical conversion, zinc-phosphate coating, deposition mechanism, titanium alloy, zinc alloy

## Abstract

Phosphate chemical conversion (PCC) technology has attracted extensive attention for its ability to regulate the surface properties of biomedical metals. However, titanium (Ti)-based alloys exhibit inertia because of the native passive layer, whereas zinc (Zn)-based alloys show high activity in acidic PCC solutions. The substrate performance affects the chemical reaction in the phosphating solution, which further leads to diversity in coating properties. In this work, the zinc-phosphate (ZnP) coatings are prepared on Ti alloy (TA) and Zn alloy (ZA) substrates using the PCC method, respectively. The coatings prepared herein are detected by a scanning electron microscope (SEM), X-ray diffractometer (XRD), laser scanning confocal microscope (LSCM), universal testing machine, contact angle goniometer, and electrochemical workstation system. The results show that the substrate performance has little effect on the phase composition but can significantly affect the crystal microstructure, thickness, and bonding strength of the coatings. In addition, the ZnP coatings improve the surface roughness of the substrates and show good hydrophilicity and electrochemical corrosion resistance. The formation mechanism of the ZnP coating is revealed using potential-time curves, indicating that the metal–solution interfacial reaction plays a dominant role in the deposition process.

## 1. Introduction

In recent years, more and more patients have bone defects caused by trauma, inflammation, congenital deformities, etc. [1]. The use of biomedical materials to repair or replace damaged tissues and organs is considered an effective means of treating bone injuries [2]. According to clinical application requirements, orthopedic implant materials are required to possess some important properties, such as biocompatibility, corrosion resistance, antibacterial activity, and suitable mechanical strength [3,4]. Metals have a long history of application in the medical field as implant materials. It is reported that metal-based materials account for about 70% of medical implants approved by the Food and Drug Administration (FDA), occupying a dominant position in clinical applications [5].

At present, titanium (Ti) and its alloys are widely applied to clinical orthopedic implants because of their good mechanical properties and biocompatibility. However, when Ti alloys are used for bone repair and replacement, whether used for a long time or only stored for a short time, they are prone to form a biofilm because of bacterial adhesion and postoperative infection [6]. Furthermore, as bioinert materials, Ti and its alloys lack active repair capabilities, resulting in the loosening of the implant due to the inability to form a long-term fusion with the surrounding tissue [7,8]. Hence, postoperative bacterial infection and poor osseointegration may directly lead to the failure of implant surgery [9], which is not conducive to long-term service. Moreover, Ti-based implants cannot be degraded and absorbed by body fluids, necessitating a secondary removal surgery, which could increase the suffering and burdens of the patients [10].

Compared with traditional biomedical materials, biodegradable metals have attracted the extensive attention of researchers. After completing the task of assisting tissue healing, the degradable implant material is gradually eroded and degraded in the physiological environment [11]. As a new generation of degradable medical metal materials, zinc (Zn) and its alloys provide a new choice for orthopedic implants [12]. Since the corrosion potential of Zn is between iron (Fe) and magnesium (Mg), its degradation rate is moderate and more in line with clinical requirements [13]. In addition, as an essential element of the human body, the Zn element plays a role in many important physiological functions. For instance, zinc ions (Zn^2+^) can regulate the differentiation of osteoblasts and osteoclasts and participate in the formation of bone growth-related hormones, thereby stimulating bone tissue growth [14]. However, Zn^2+^ has a dual effect on osteoblasts, as low concentrations can improve cell viability, while high concentrations have the opposite effect [15]. Excessive release of Zn^2+^ during the degradation process of Zn-based alloys can lead to severe cytotoxicity [16]. Therefore, the current focus of functionalized surface-modification technology on Zn alloys points to controlling the corrosion rate and improving their biocompatibility and antibacterial properties [16,17].

The phosphate chemical conversion (PCC) method has a long history of application in the field of corrosion protection of metal materials and has been gradually used in the preparation of surface-modification coatings for biomedical materials in recent years [18]. During the process of coatings preparation, some specific metal elements with antibacterial activity and osteogenic immunomodulatory functions can be introduced. Then, the biocompatibility and chemical stability of implant materials can be improved by adjusting the phase composition and morphology of the coatings. With stable chemical properties and good biocompatibility, zinc-phosphate (ZnP) coatings have broad application prospects in the biomedical field [16,19]. ZnP coatings with various microstructures and functional compositions can improve the antibacterial and biological activity of the metals. In addition, a ZnP coating also can regulate the degradation rate of Zn alloys and improve the cytocompatibility, hemocompatibility, and antibacterial properties by slowing the release of Zn^2+^ [5,16].

Ti-based alloys are biologically inert because of the native passive layer, whereas Zn-based alloys show high activity in acidic PCC solutions. Due to their different surface properties, the coating formation mechanism of the PCC coating corresponding to the two substrates is essentially different, which directly determines the coating quality. In this study, we focus on the effect of substrate performance on the microstructure and properties of the ZnP coating. The ZnP coatings are prepared on Ti alloy (TA) and Zn alloy (ZA) surfaces by the PCC method, respectively. The phase composition, microstructure, and element composition of the ZnP coatings are studied. The comparison of coating thickness, bonding strength, surface roughness, wettability, and electrochemical corrosion behavior is systematically researched. In addition, with the help of the potential-time curves immersed in the phosphate solution, the formation mechanism of the ZnP coating on TA and ZA surfaces is explored.

## 2. Experimental

### 2.1. Coating Preparation

Commercial TA (Grade TA2) and ZA (Zn-1.0; Mg-0.5 Ca) specimens with a disk shape (ϕ10 × 3 mm) acted as substrates. After being sanded with #180, #600, and #1000 grit sandpaper in turn, the samples were ultrasonically cleaned with acetone, ethanol, and deionized water, respectively. The chemical compositions (Table 1) were sequentially added to obtain the phosphating solution, and then the pH was adjusted to 2.5 with sodium hydroxide (NaOH, 10 mol/L). To overcome the influence of the oxide film on the TA surface, a galvanic coupled system was assembled by clamping each TA plate with a pure iron (Fe) alligator clip to assist the coating growth on TA. The prepared substrates were immersed in the Zn-P bath at 25 °C for 30 min to obtain the ZnP coating. After the conversion reaction, the ZnP-coated samples were cleaned and dried for subsequent detection.

### 2.2. Coating Characterization

The microstructure and thickness of the ZnP coating were observed through a field-emission scanning electron microscope (FE-SEM, SU-70, Hitachi, Tokyo, Japan). An energy-dispersive spectrometer (EDS) was utilized to analyze the elemental composition of the coating. The phase composition of the coating was examined by an X-ray diffractometer (XRD, DMAX-2500PC, Rigaku, Tokyo, Japan), which detected between 5° to 90° with a scanning speed of 10° min^−1^.

### 2.3. Tensile Adhesion Tests

To identify the bonding strength of the ZnP coatings on TA and ZA substrates, tensile adhesion tests were carried out according to the ASTM C633-01 standard. Before testing, the coated samples were bonded between two parallel aligned stainless-steel cylinders using acrylic adhesive and then cured at room temperature for 24 h. The tests were performed using a universal testing machine (WDW-5, STAR, Jinan, China), equipped with a 5 kN load–cell capacity. The vertical tensile load was applied to the samples with a constant rate of 1 mm min^−1^. The ratio of the maximum tensile load to the sample surface area was considered the bonding strength.

### 2.4. Surface Roughness and Wettability

The three-dimensional (3D) topography, surface average roughness (Sa), and root mean squared roughness (Sq) of specimens were measured via a laser confocal scanning microscope (LSM 800, Zeiss, Oberkochen, Germany) on a surface area of 600 μm × 600 μm. The contact angle (θ) was detected by the sessile drop technique, and water droplets (1 μL) were dropped onto the surface of the specimen via a micro-syringe, followed by recording the static θ values via a contact angle goniometer (DSA100S, KRUSS, Hamburg, Germany).

### 2.5. Electrochemical Measurements

The electrochemical behaviors of the bare and coated samples were tested by an electrochemical workstation (CHI660e, Shanghai, China). The three-electrode cell, which contains a saturated calomel electrode (SCE, reference electrode) and a platinum (Pt) sheet (counter electrode), and the tested samples (working electrode) were used to carry out the detection in a 0.9 wt.% NaCl solution at 25 ± 1 °C. The open circuit voltage (OCP) was established before the electrochemical impedance spectroscopy (EIS) testing. The EIS was performed with a sinusoidal voltage perturbation amplitude of 5 mV and a frequency range of 100 kHz to 0.01 Hz. The polarization curve was carried out with a scanning rate of 1 mV/s, which determines the corrosion potential (E_corr_), corrosion current density (I_corr_), and anode/cathode Tafel slope (β_a_/β_c_). In addition, the polarization resistance (R_p_) was calculated with the following Equation (1).

(1) $$R_{p}=\frac{\beta_{a}\cdot \left|\beta_{c}\right|}{2.303\cdot I_{\mathrm{corr}}\cdot {(\beta }_{a}+{|\beta }_{c}|)}$$

The corrosion rate P_i_ (mm/year), which is related to the I_corr_ (mA/cm^2^), can be calculated using Equation (2) [20].

(2) $$P_{i}=22.85\cdot I_{\mathrm{corr}}$$

The potential-time curve was recorded to explore the coating formation mechanism via monitoring the open circuit potential (OCP) of the samples in the ZnP bath at 25 °C from the start of immersion to 1800 s.

## 3. Results and Discussion

### 3.1. Phase Composition

**Figure 1:**
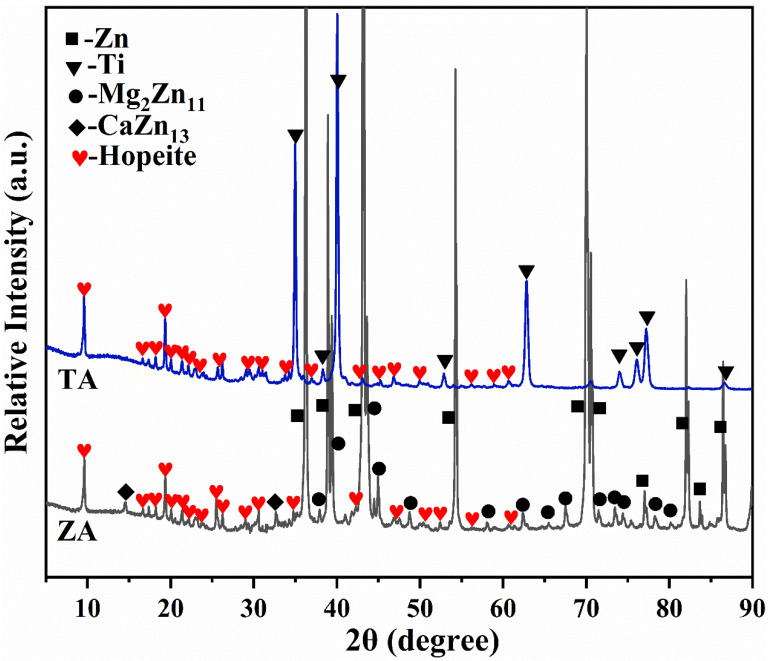
XRD patterns of the ZnP-coated TA and ZA samples.

The XRD patterns of the coating on metal substrates, treated in the Zn-P bath, are shown in Figure 1. It displays that the bare TA substrate shows diffraction lines ascribed to the Ti phase only. For the ZA substrate, the diffraction peaks of the typical intermetallic compounds Mg_2_Zn_11_ and CaZn_13_ were detected besides the Zn single phase, which revealed the addition of trace Mg and Ca elements from the Zn-Mg-Ca system [21,22]. Especially, the phase composition of coatings on both TA and ZA substrates was dominated by hopeite (Zn_3_(PO_4_)_2_·4H_2_O), which indicates that the substrate performance had little effect on the phase composition of the coating. As a typical bio-inert metal, the TA cannot participate in the conversion reaction, thus the coating prepared on its substrate belongs to the deposition conversion coating. In the coating preparation process, the film-forming ions (such as Zn^2+^, HPO_4_^2−^, PO_4_^3−^ ions, etc.) were all provided by the Zn-P conversion bath. After the ions in the solution interacted and reached supersaturation, ZnP crystals were precipitated on the surface of the TA. On the contrary, since ZA exhibits high activity in acidic solutions, the self-corrosion process occurs rapidly, as the ZA substrate is immersed in the conversion solution and large amounts of Zn^2+^ are released simultaneously. Zn^2+^ acts as film-forming ions to involve the ZnP coating formation, thus self-corrosion process has little effect on the phase composition of the coating [22].

### 3.2. Microstructure of the Coatings

**Figure 2:**
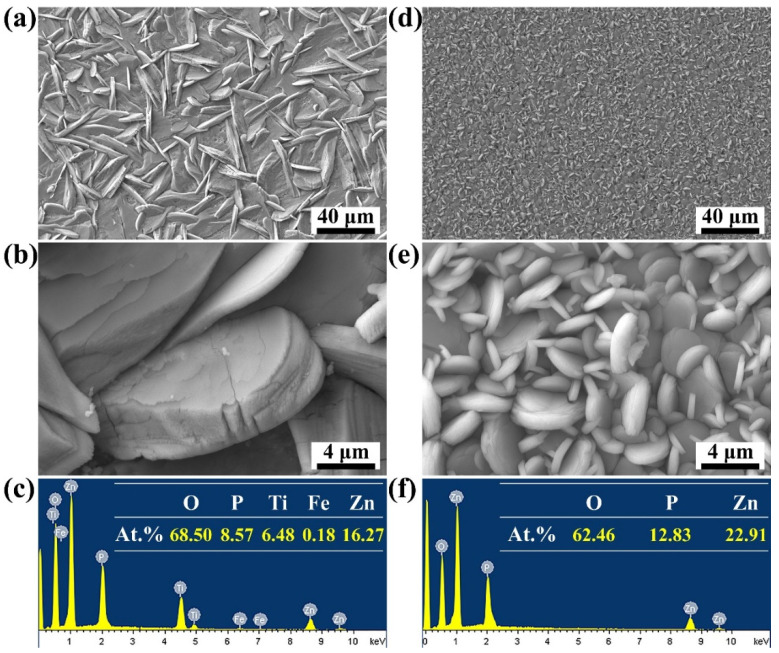
The FE-SEM morphologies and elemental composition of ZnP coating on TA (**a**–**c**) and ZA (**d**–**f**).

Figure 2 depicts the surface morphologies and microstructure of the ZnP coating obtained on the TA and ZA substrates. Full coverage of the ZnP coating was achieved on both surfaces of the metal without exposing the substrate. However, with the change in metal substrate performance, the morphology of the ZnP crystals varied greatly. Specifically, plate-like crystals with a size of about 10–40 μm were grown and attached to the surface of TA samples after Zn-P bath treatment (Figure 2a). The corresponding high-magnification image shows that the thickness of the crystals is about 1–4 μm (Figure 2b). By contrast, the ZnP crystals formed on the ZA surface exhibited a typical disk-like morphology with a size ranging from 1 to 4 μm (Figure 2d). The initially formed crystals of about 2–4 μm overlapped each other and formed a dense underlying coating. Subsequent finer crystals (1–2 μm) were randomly interspersed and embedded in the underlying crystals (Figure 2e). These changes in crystal microstructure and morphology were closely related to the performance of the metal substrate. In particular, the substrate performance affected the chemical reaction in the phosphating solution, which further led to differences in the microenvironment around the metal. Correspondingly, the nucleation and growth rate of crystals also showed significant differences. Because the ZA participated in the conversion reaction, a large amount of Zn^2+^ was released and dispersed on the metal surface, which could be regarded as the nucleation site of the coating crystals. Various ions in the solution near the surface of the substrate migrated rapidly to the Zn^2+^ escape point, where they combined and crystallized. With the number of crystals and the formation rate increase, the growth space of the crystals was suppressed, causing smaller crystal sizes and a close arrangement [23]. However, the formation of the coating on TA substrate is considered a deposition process [23]. During this process, various ions dispersed in the Zn-P solution underwent conversion reactions and reached supersaturation, and then the insoluble ZnP began to precipitate and deposit on the surface of the chemically stable TA substrate. Crystals could fully expand to larger sizes due to sufficient growth space in the solution. Ultimately, a continuous and complete ZnP coating that tightly wraps the substrate was formed.

The elemental compositions of ZnP coatings with different microstructures are shown in Figure 2c,f. The energy peaks of O, Zn, and P appeared in the conversion coatings on TA and ZA. Besides, the atomic ratio of Zn/P assessed in this analysis is close to the theoretical atomic ratio of hopeite (Zn_3_(PO_4_)_2_·4H_2_O). The above results show that the coatings with different microstructures were all composed of hopeite, which is consistent with the results of XRD.

### 3.3. Coating Thickness and Bonding Strength

**Figure 3:**
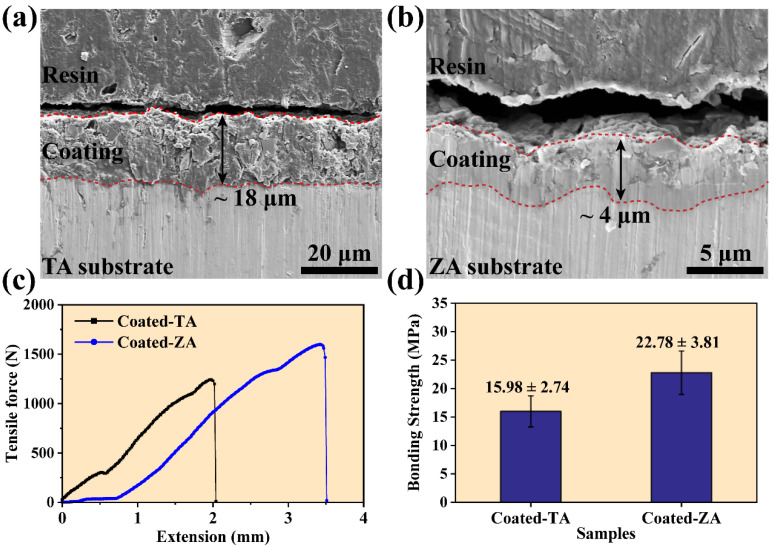
The cross-sectional FE-SEM micrographs and thickness of the ZnP coatings on (**a**) TA and (**b**) ZA samples, (**c**) tensile-extension curves, and (**d**) bonding strength variation of ZnP coatings on TA and ZA samples.

The cross-section morphologies and the corresponding thickness of the ZnP coating on the TA and ZA substrates are depicted in Figure 3a,b. According to the cross-sectional morphology, there was no obvious crack between the coating and substrate, indicating that the interface between the coating and substrate was well combined. The results show that the thickness of the coating on TA (18 μm) was 4 times larger than that of the coating on ZA (4 μm), which may be attributed to the difference in the coating microstructure. Excluding the effect of composition, the thickness was proportional to the crystal size [24]. As mentioned earlier, the performance of the substrate affected the conversion microenvironment, which ultimately had a significant impact on the crystal size and arrangement. Since the self-etching process of ZA provided more growth sites, the crystal had a faster growth rate, which led to the reduction of crystal size. As shown in Figure 2, the size of the plate-like ZnP crystals on the TA was significantly larger than that of the disk-like crystals on the ZA substrate, thus the thickness of the coating on TA increased correspondingly.

The results of tensile adhesion tests are shown in Figure 3c,d. The bonding strength of the ZnP coating on TA was about 15.98 ± 2.74 MPa. As a comparison, the value of the coating on ZA increased to 22.78 ± 3.81 MPa. This result is mainly caused by the difference in substrate and coating thickness. The ZA substrate could participate in the growth of the ZnP coating through self-corrosion, and it was chemically bonded with the surface coating. In contrast, the growth of the coating on TA substrate relied on the deposition of ZnP crystals in solution. In addition, the thickness and crystal size of the coatings also had a significant effect on the bonding strength. The thickness of the ZnP coating with larger crystals on the TA substrate was about 18 μm, which makes the coating prone to failure during the tensile process, while the smaller thickness and finer crystal size of the coating on ZA had a significant contribution to improving the bonding strength.

### 3.4. Surface Roughness and Wettability of the Coatings

**Figure 4:**
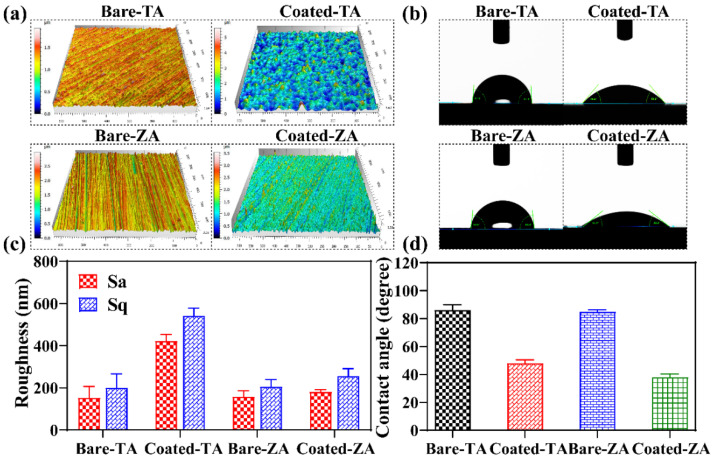
(**a**) LSCM 3D profile micrographs, (**b**) digital images of the water droplets, quantitative measurement results of (**c**) surface roughness, and (**d**) contact angles (θ) of the ZnP coatings on TA and ZA samples.

Roughness and wettability, as important properties of the material surface, are directly involved in influencing the interaction of the implanted material with the surrounding physiological environment [25]. Figure 4a depicts the 3D profile micrographs of the ZnP coating on the TA and ZA substrates. The corresponding roughness values (S_a_, S_q_) are presented in Figure 4c. Both TA and ZA substrates show parallel scratch grooves, which are formed by grinding during sandpaper pretreatment. Compared with the bare substrates, the ZnP-coated samples presented an irregular peak–valley distribution because of the disorderly arrangements of the crystals. The quantitative calculation results show that the roughness values of both TA and ZA substrates were lower than that of the corresponding ZnP-coated samples. As shown in Figure 2, the ZnP crystals on the surface of TA had a large size range and were dispersed disorderly, leading to the largest roughness value. However, the roughness of the coated-ZA samples was approximately equal to that of the bare-ZA substrate, which was due to the smaller crystal size of the coating on the ZA substrate and the uniform distribution of crystals. In addition, slight scratch grooves could be observed on the coated-ZA samples, proving a small value of the coating thickness, which is consistent with the coating thickness results.

Figure 4b,d depicts the digital images of water droplet morphology and contact angle (θ) calculation results obtained from the wettability testing of bare and coated samples. The wettability of the material surface directly affects the biological properties by inducing cell adhesion and differentiation [26]. Compared with the bare TA and ZA substrates, the coated samples had significantly low contact values (θ), showing that the ZnP coating could effectively improve the wettability of the metal surfaces. The wettability of the coating mainly depended on its surface chemical composition and microstructure [19]. The dominant phase of the coatings on both TA and ZA was hopeite, which has good hydrophilicity [19]. The improved hydrophilicity of the coated-ZA is mainly attributed to its small crystal size and the existence of gaps between the single crystal grains. Besides, the densely arranged ZnP coating on the TA surface exhibit obvious morphology and structural fluctuations (Figure 4a). The contact angle value was inversely proportional to the roughness [27], while the above-mentioned crystal features significantly increased the roughness of the coating. Therefore, the hydrophilicity of the coated-TA samples shows visible improvement.

### 3.5. Corrosion Resistance

**Figure 5:**
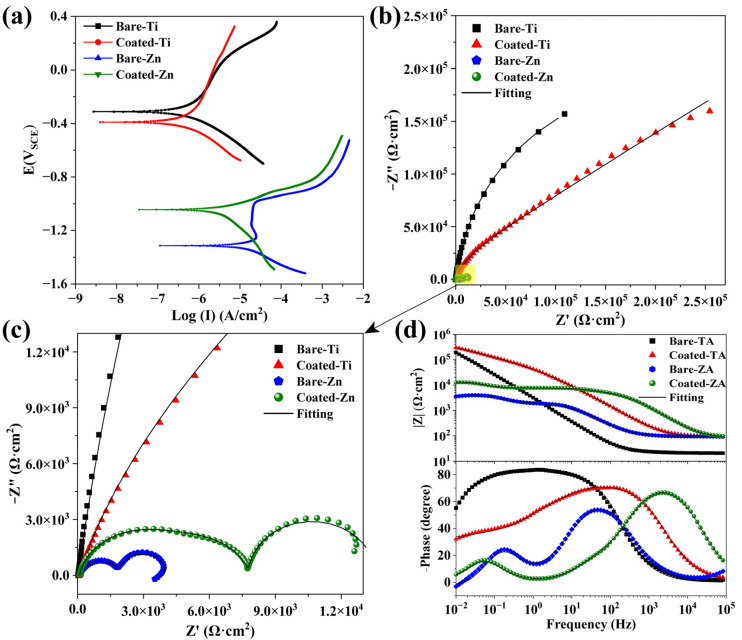
The electrochemical performance tests of the ZnP coatings on TA and ZA substrates. (**a**) Potentiodynamic polarization curves and impedance spectra present in (**b**–**d**) Bode and phase-angle plots.

Figure 5a presents the potentiodynamic polarization curves of the substrate and ZnP-coated samples, and the electrochemical data calculated by the Tafel fit are summarized in Table 2. In general, a greater value of corrosion potential (E_corr_) and lower value of corrosion current (I_corr_) result in better corrosion-protection performance [28]. By comparing the polarization curves of the two kinds of substrate, the corrosion resistance of the TA was better than that of the ZA substrate, which is explained by the difference in the electrode potential of the metal. Then, the bare and ZnP-coated TA and ZA substrates were analyzed separately for comparison. The strong affinity between TA and oxygen in the air promotes a spontaneous reaction to form a stable oxide layer (mainly composed of TiO_2_) [29]. The TiO_2_ oxide layer still maintained chemical stability with a short immersion time in 0.9% NaCl solution. As shown in Figure 5a, the ZnP coating exhibits a significant effect on the corrosion behavior of the TA substrate. Specifically, the I_corr_ of the coated-TA is shifted to the negative direction, accompanied by the decreasing E_corr_. In particular, since the I_corr_ has a linear relationship with the P_i_, the decreasing I_corr_ can effectively indicate the improvement of the corrosion resistance [30]. Correspondingly, the coated-TA exhibits the largest R_p_ value, indicating its excellent corrosion resistance. The electrochemical data of the I_corr_, E_corr_, and P_i_ in Table 2 support the above analysis for the polarization curves.

Due to the formation of a natural oxide layer in the air, an obvious pseudo-passivation zone appears on the anodic polarization curve of the bare-ZA substrate [31,32]. By contrast, the curve of the coated-ZA samples shows a shift in the direction of positive E_corr_ and negative I_corr_. Higher E_corr_ indicates lower corrosion probability, whereas higher I_corr_ represents a faster corrosion rate [33,34]. As shown in Table 2, the I_corr_ value of the bare-ZA is one order of magnitude higher than that of the coated-ZA sample. In addition, after being protected by ZnP coating, the R_p_ value of the ZA substrate is increased by more than seven times. Besides, the P_i_ calculation results also show that the corrosion rate of bare-ZA is nearly ten times faster than that of the coated-ZA samples. All of these results prove that the ZnP coating with relatively higher corrosion resistance can regulate the corrosion behavior of the ZA substrate.

**Figure 6:**
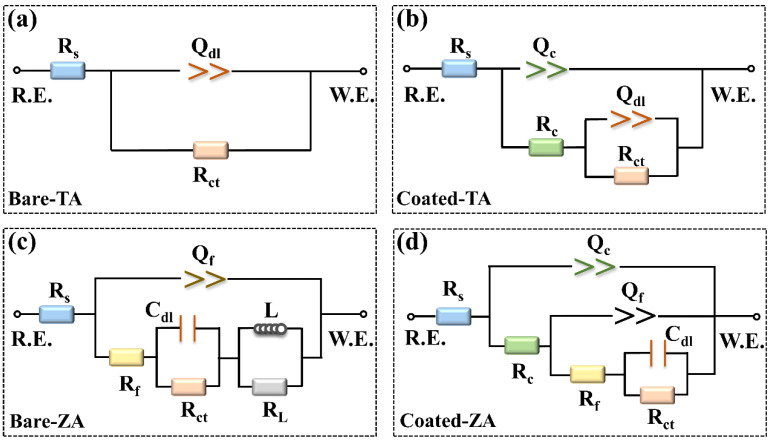
The equivalent electrical circuits are used to fit the impedance behaviors of (**a**) Bare-TA, (**b**) Coated-TA, (**c**) Bare-ZA, and (**d**) Coated-ZA. R.E.: reference electrode, W.E.: working electrode.

To further analyze the electrochemical corrosion resistance of the bare and ZnP-coated samples, EIS measurements were carried out. Figure 5b,c depicts the Nyquist plot of the samples in the EIS tests. According to the results, both the bare and coated TA samples exhibit incomplete semicircular arcs, while the latter possess larger-capacitance loop diameters, indicating the better corrosion resistance of the ZnP coating [35]. Figure 5d displays the Bode magnitude and phase-angle plots, indicating that the bare-TA and coated-TA samples exhibit different relaxation features. The bare-TA substrate only contains one time constant, but the coated-TA has two capacitive loops, which represent the presence of two interfaces (solution/coating and coating/substrate) in the coated samples [35,36]. In particular, the capacitive loop at high frequency corresponds to the ZnP coating, but the capacitive loop at low frequency is related to the double layer at the interface between the substrate and solution. Based on the difference between the EIS plots of the bare and coated TA samples, the impedance data are fitted by two equivalent electrical circuits (EEC, (Figure 6a,b), respectively).

As for the bare and coated ZA samples, three distinct humps shown in the Bode phase-angle plot correspond to the three relaxation processes. The first time constant is located at a high frequency, which is due to the corrosion product generated after the ZA substrate is immersed in the solution. The second time constant at mid-frequency is attributed to the double layer between the substrate and the solution interface, and the third time constant at low frequency corresponds to the inductive reactance arc. For the coated-ZA samples, the time constant at high frequency arises from the ZnP coating, while the time constant at mid-frequency is associated with the corrosion product. Besides, the corrosion product and substrate exist in the low-frequency time constant. The diameter of the capacitive arc reflects the corrosion resistance of the specimen. Compared with the ZA substrate, the ZnP-coated specimens possess a larger capacitance arc diameter, which further indicates the effective protective ability of the ZnP coating.

The electrical equivalent circuits in Figure 6 are employed to fit the EIS spectra, and the corresponding fitted results are shown in Table 3. In the EEC model, R_s_ represents the solution resistance. R_c_ and R_ct_ are the resistance of the ZnP coating and charge transfer, while Q_c_ and Q_dl_ are used to describe the constant phase element (CPE) of the ZnP coating capacitance and the electric double-layer capacitance [35]. The pure capacitive element (C) is replaced by the circuit element Q to account for the capacitance deviation caused by the uneven surface of the coating [37], which is expressed as Q = Cωn^−1^. Among them, ω refers to the angular frequency, and n represents the fractional index of the CPE that reflects the deviation from the ideal capacitance (0 ≤ n ≤ 1, n = 1 corresponds to the behavior of the pure capacitance [38]). Moreover, except for the relevant components of the ZA samples, R_f_ and Q_f_ refer to the capacitive arc associated with the dielectric properties of the corrosion product, and R_f_ and Q_f_ are the resistance and constant phase elements of the corrosion product, respectively. L and R_L_ are inductance and inductance resistance, which shows the relaxation of the adsorbate that is connected with Zn^2+^ and/or Zn(OH)_2_ generated on the ZA surface [22,39]. The fitting results corresponding to the ECC models of the bare and ZnP-coated samples are in good agreement with the tested results (χ^2^ < 0.01), as shown in Table 3. The sum of R_c_ and R_ct_ is an important indicator for predicting the overall corrosion resistance. According to the results, the sum of the resistance of the TA substrate is much higher than that of the ZA substrate. In addition, compared to the substrate, the sum of the resistance of the ZnP-coated samples is increased, showing similar effects in both TA and ZA samples. This result means that the ZnP coating provides effective protection, allowing the adjustment of the bare substrate corrosion resistance.

### 3.6. Formation Mechanism of the PCC Coating

**Figure 7:**
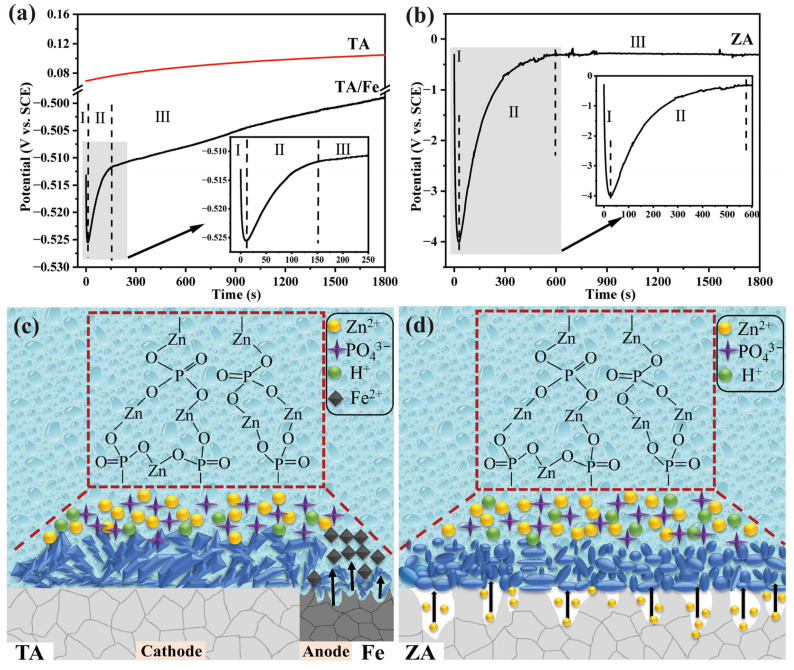
The potential-time curves of (**a**) TA and (**b**) ZA during the coating deposition in the phosphate chemical-conversion solution and a schematic diagram of the ZnP coating formation on (**c**) TA and (**d**) ZA substrates.

The growth of the PCC coating is an artificially induced and controlled process, which is described as the crystal growth of metal phosphate on the surface of the substrate after reaching the supersaturated state in the conversion solution [18]. As the coating growth contributes to the potential change of the metal surface, monitoring the OCP is considered an effective means to reveal the coating growth process. Figure 7a,b show the potential-time (E(t)) curves from the start of immersion to 1800 s of TA and ZA substrates obtained by the OCP test. Specifically, the change in potential is affected by the formation of the coating, while the plateau region signifies that the chemical conversion has reached a stable process. As shown in Figure 7a, the bare TA and coupled TA substrates exhibit different E(t) curves. In detail, the E(t) curve of the bare TA substrate has no obvious fluctuation, especially in the initial stage, indicating that there is no coating formation process on its surface. According to the Pourbaix diagram of the TA [40], titanium ions (Ti^2+^) cannot be released into the solution within the initial potential (about 0.07 V). Therefore, the TA substrate has no corrosion behavior in the PCC solution, and the formation of the ZnP coating is a deposition process driven by solution supersaturation [23]. After forming a coupled system with Fe, the TA exhibits a significantly different E(t) curve, which can be described in three steps:

**Stage I.** Electrochemical corrosion dissolution of anodic Fe. Based on our previous work, the galvanic couple (TA/Fe) induction plays a key role in the phosphate coating preparation on inert metals [19,41]. Specifically, the dissolution of Fe occurs due to the potential difference between the two coupled materials (Equation (3)), resulting in a hydrogen evolution reaction on the cathodic TA (Equation (4)). The severe corrosion of the anodic Fe at this stage leads to a sharp decrease of the potential in the E(t) curve.

Fe − 2e^−^ → Fe^2+^ (3)

2H^+^ + 2e^−^ → H_2_ ↑ (4)

**Stage II.** The rapid growth of the coating on anodic Fe together with initial nucleation deposition on cathodic TA. The above-mentioned hydrogen evolution reaction results in an increase in pH at the metal/solution interface, which promotes the further ionization of phosphate at the interface (Equation (5)). This process makes the solution reach a supersaturated state, and then crystal nuclei are deposited on cathodic TA (Equation (6)). Meanwhile, since Fe also has a micro-anode and a micro-cathode, the coating also grows rapidly on its surface (Equation (7)). The E(t) curve at this stage shows a rapid rise in potential due to the non-conductivity of the phosphate coating.

H_2_PO_4_^−^ ↔ HPO_4_^2−^ + H^+^ ↔ PO_4_^3−^ + 2H^+^ (5)

3Zn^2+^ + 2H_2_PO_4_^−^ +2H^+^ + 4H_2_O + 6e^−^ → Zn_3_(PO_4_)_2_·4H_2_O↓ + 3H_2_↑ (6)

Fe^2+^ + 2Zn^2+^ +2PO_4_^3−^ + 4H_2_O → Zn_2_Fe(PO_4_)_2_·4H_2_O ↓ (7)

**Stage III.** Slow deposition of crystals on the cathodic TA and the formation of the coating. After the anodal Fe is covered with the coating, the potential difference between the TA/Fe decreases, and the coating formation rate on the cathodic TA slows down; thus the E(t) curve at this stage shows a slow increase in the potential.

Unlike the TA, the self-corrosion of the ZA substrate in the phosphating solution can accelerate the coating formation process. As shown in Figure 7b, the E(t) curve of the ZA shows a similar trend to TA/Fe. Correspondingly, it is also defined in three steps: (I) dissolution of the ZA substrate, (II) coating formation, and (III) dynamic balance. In the first stage, the initial potential of the ZA formed in the phosphating solution is about −0.5 mV. According to the Pourbaix diagram of ZA [40], this initial potential allows the ZA to release Zn^2+^ through self-corrosion (Equation (8)). Therefore, the potential shows a sharp decrease in the first 25 s. Similar to TA/Fe, phosphate crystals begin to precipitate on the ZA after the potential rapidly reaches the minimum value, as shown in Equation (6). This process, in which the potential gradually increases, continues for about 600 s until a continuous coating is formed on the ZA. After that, the coating on the ZA prevents direct contact between the phosphating solution and the substrate, and the coating growth reaches the dynamic equilibrium stage.

Zn → Zn^2+^ + 2e^−^ (8)

Shematic diagrams of the ZnP coating growth on different substrates are shown in Figure 7c,d. With the help of the E(t) curves, it is demonstrated that the metal–solution interfacial reaction plays a dominant role in the coating formation process. The self-corrosion of the ZA can generate more nucleation points on its surface, and the corrosion reaction also provides the initial driving force for nucleation. These factors result in the accelerated growth of the coating on the ZA, which in turn affects the reduction of crystal sizes and coating thickness. By contrast, the coating growth on the TA depends on the TA/Fe coupling system, which leads to a slower coating growth rate and a significant increase in crystal size.

## 4. Conclusions

In this work, ZnP coatings based on the hopeite phase were constructed on TA and ZA substrates. It was identified that the change in the metal substrate had little impact on the phase composition but a great influence on microstructure and properties for the ZnP coatings prepared by the PCC method. Compared with the plate-like ZnP coating on the surface of the TA, the higher chemical activity of ZA results in the crystal appearing with a disk-like morphology, thereby reducing the roughness and improving the hydrophilicity. Compared with TA, the ZnP on ZA has a smaller thickness and greater bonding strength. Electrochemical corrosion results reveal that the ZnP coating has good corrosion resistance, which plays a significant role in the corrosion protection of the substrate. The coating formation mechanism on TA and ZA substrates was analyzed by the potential-time curves, and the results demonstrate that the metal–solution interface reactions dominate the deposition process.

## Figures and Tables

**Table 1:** Chemical compositions of the phosphating solution and treatment conditions.

Coating Type	Bath Composition	Concentration	Conditions
Phosphating	ZnO	25 g/L	pH = 2.50 T = 25 °C t = 30 min
	HNO_3_	30 mL/L	
	H_3_PO_4_	10 mL/L	
	NaClO_3_	2 g/L	
	Ca(NO_3_)_2_·4H_2_O	5 g/L	
	C_6_H_8_O_7_·H_2_O	5 g/L	

**Table 2:** The electrochemical parameters determined by the polarization curves of bare and ZnP-coated TA and ZA samples. Values are shown as mean ± SD, n = 3.

Sample	E_corr_ (V)	I_corr_ (×10^−6^, A/cm^2^)	β_a_ (V·dec^−1^)	−β_c_ (V·dec^−1^)	R_p_ (KΩ·cm^2^)	P_i_ (mm/Year)
Bare-TA	−0.29 ± 0.02	0.50 ± 0.04	0.24 ± 0.01	0.15 ± 0.01	83.33 ± 8.32	0.012 ± 0.003
Coated-TA	−0.36 ± 0.02	0.41 ± 0.08	0.23 ± 0.03	0.16 ± 0.01	101.97 ± 24.81	0.009 ± 0.002
Bare-ZA	−1.31 ± 0.01	43.46 ± 5.94	0.99 ± 0.17	0.19 ± 0.02	1.59 ± 0.13	0.993 ± 0.111
Coated-ZA	−1.05 ± 0.02	4.64 ± 0.31	0.11 ± 0.01	0.28 ± 0.02	7.36 ± 0.88	0.106 ± 0.006

**Table 3:** EIS fitted parameters of the equivalent electrical circuits for bare and ZnP-coated TA and ZA samples. Values are shown as mean ± SD, n = 3.

Samples	Bare-TA	Coated-TA	Bare-ZA	Coated-ZA
R_s_ (Ω·cm^2^)	83.19 ± 2.91	92.15 ± 3.51	94.20 ± 1.27	85.78 ± 2.50
Q_c_ (×10^−6^ Ω^−1^·cm^−2^·S^−n^)	-	3.13 ± 0.11	-	0.31 ± 0.03
n_c_	-	0.88 ± 0.03	-	0.90 ± 0.01
R_c_ (KΩ·cm^2^)	-	35.68 ± 5.03	-	4.84 ± 0.62
Q_dl_ (×10^−5^ Ω^−1^·cm^−2^·S^−n^)	6.01 ± 0.19	0.95 ± 0.02	-	-
n_dl_	0.92 ± 0.01	0.43 ± 0.05	-	-
Q_f_ (×10^−5^ Ω^−1^·cm^−2^·S^−n^)	-	-	1.69 ± 0.02	0.47 ± 0.07
n_f_	-	-	0.87 ± 0.02	0.80 ± 0.07
R_f_ (KΩ·cm^2^)	-	-	1.02 ± 0.17	2.96 ± 0.67
C_dl_ (10^−4^ F·cm^−2^)	-	-	5.09 ± 1.42	5.93 ± 0.65
R_ct_ (KΩ·cm^2^)	422.80 ± 30.97	4298.00 ± 9.90	2.30 ± 0.08	6.22 ± 0.71
L (×10^3^ H·cm^−2^)	-	-	6.59 ± 0.46	-
R_L_ (KΩ·cm^2^)	-	-	0.81 ± 0.08	-
χ^2^ (10^−3^)	1.00 ± 0.02	0.29 ± 0.04	1.17 ± 0.30	0.18 ± 0.01

## Data Availability

The data presented in this study are available on request from the corresponding author. The data are not publicly available due to issues related to the proprietary rights.

## Sample Availability

Samples of the compounds are not available from the authors.
